# Interpreting Functional Recovery and Return to Work After Full Endoscopic Lumbar Discectomy

**DOI:** 10.1177/21925682261506611

**Published:** 2026-11-02

**Authors:** Yuxiang Lan, Changkun Zheng

**Affiliations:** 1 47858 Fujian University of Traditional Chinese Medicine, Fuzhou, China; 2 Department of Orthopedics, 599390 Fuzhou Second General Hospital, Fuzhou, China

To the Editor,

We read with interest the randomized trial by Sharma et al comparing full endoscopic discectomy (FED) with tubular microscopic discectomy (TMD) for lumbar disc herniation.^1^ Two points merit clarification to support interpretation of the reported recovery benefits. First, Table 3 reports final Oswestry Disability Index (ODI) scores of 6.3 and 8.8, respectively (P < .0001). Specifying whether these values represent raw or normalized scores would clarify the scale of the 2.5-unit difference. The clinical importance of this difference would be easier to assess with a between-group effect estimate and its 95% confidence interval. Reporting the proportions achieving a justified threshold for meaningful individual improvement would provide complementary information. Thresholds for individual change and between-group differences address distinct questions and should be interpreted separately.^2^ Together, these measures would help readers assess the magnitude, precision, and clinical relevance of the functional advantage.

Second, clarification of the return-to-work results would help establish the consistency of the reported recovery advantage. Table 6 places all 112 FED and 90 TMD participants in categories spanning 4–8 weeks, with respective means of 4.93 and 6.00 weeks. Supplementary Table S3, however, reports corresponding means of 2.63 and 3.73 weeks for the single-level subgroups. If timing and definitions were identical, these subgroup means would fall below the earliest return-to-work category listed in Table 6. Clarifying the time origin, units, and analysis denominators would help reconcile these findings. Further context on baseline employment, occupational demands, and preoperative sick leave would also aid interpretation. Manual work and prolonged preoperative sick leave have been associated with delayed return after lumbar disc surgery.^3^ Defining whether return to work meant resuming any duties or usual duties, and describing postoperative work advice, would improve the outcome’s applicability. These details would strengthen the basis for counseling patients about recovery after FED and TMD.

## Data Availability

No new datasets were generated or analyzed for this Letter; the discussion concerns the published article and its supplementary material.*
